# Formula-feeding practice and associated factors among urban and rural mothers with infants 0–6 months of age: a comparative study in Jimma zone Western Ethiopia

**DOI:** 10.1186/s12887-019-1789-8

**Published:** 2019-11-04

**Authors:** Lakew Abebe, Mamusha Aman, Shifera Asfaw, Hailay Gebreyesus, Mebrahtu Teweldemedhin, Abebe Mamo

**Affiliations:** 1 Departments of Health Education and Behavioral Sciences, Jimma University, Jimma, Ethiopia; 2 Department of Public Health, College of Health Sciences, Aksum University, P.O. Box 298, Aksum, Ethiopia; 3 Department of Medical Laboratory Science, College of Health Sciences, Aksum University, Aksum, Ethiopia

**Keywords:** Formula-feeding practice, Predictors of formula feeding, Jimma, Ethiopia

## Abstract

**Background:**

Infants are in a state of rapid development and maturation; the growth rate is most rapid during the first 4 to 6 months of life. Few studies indicated that in developing countries including Ethiopia the prevalence and duration of breastfeeding is declining and being replaced by formula milk. Therefore, this study aimed to assess the formula-feeding practice and its associated factors among urban and rural mothers with infants 0–6 months of age in the Jimma Zone, Western Ethiopia.

**Methods:**

A community-based cross-sectional study was conducted from November 7, 2015, to January 10, 2016, in the Jimma Zone. The quantitative data were collected from a sample of 714 respondents using a multistage sampling technique. Data were collected through a structured questionnaire and the multivariate logistic regression model was used to show predictors of the formula-feeding practice among mothers with infants 0–6 months of age.

**Result:**

The proportion of mothers who feed their baby formula-based was 47.2%, of which 34.5% were living in rural areas and 65.5% were living in urban areas. Among the mothers living in urban areas, the likelihood of formula-feeding was significantly associated with maternal educational status and attitude towards formula-feeding. On the other hand, being attended by relatives/friends and the traditional birth attendant was significantly associated with the formula-feeding practice among mothers who live in rural areas.

**Conclusion:**

Nearly half of the mothers in the study area practice formula-feeding for their infant. Therefore, sustained community based nutritional health education is recommended for pregnant and lactating mothers to reduce the practice of formula-feeding for infants.

## Background

The neonatal period represents one of the most critical and vulnerable periods in human life, particularly with respect to nutrition [1]. The newborns are in a state of rapid development and maturation, because the growth rate is most rapid during the first four to 6 months of life. Demands the availability of essential nutrients [2, 3].On the other hand, an infant’s tolerance for deviations in food intake is limited because most of the organs that play an essential role in metabolism and its regulation are immature. Other organs such as the central nervous system are also in a process of intensive development and maturation [4–6].

Nutritional inadequacy during the infant period causes prolonged and sometimes irreversible effects on the growth and development and adult physiological function [7]. Another factor which gives rise to specific nutritional problems during this period is that the tendency of children to be monotonous; lacking which results nutritional insufficiency if [8, 9]. However, several studies in developing countries indicated that the prevalence and duration of breastfeeding are declining and are being replaced by formula-feeding including plain water, butter, fruits juice and other local foods, while colostrum is discarded as unclean [10, 11]. These are the families who cannot afford the high cost of formula feeding [11]. A study conducted in Nigeria among children aged below 6 months showed that the proportion of infants who were given formula feeding was 83.6%, [12]. Other similar studies conducted in India, Nepal, and Bangladesh revealed that more than half of the mothers with a child aged below 1 year were formula -feeding their babies [13–15]. Also, in 11 rural villages in Northern India formula –feeding was found to be associated with higher morbidity and mortality compared to children who were breastfed by their mother [13].

Formula-feeding results in many health problems, as it has often led to an increased incidence of childhood conditions. Diarrhea, malnutrition, acute respiratory infection, protein-energy malnutrition, iron deficiency (which leads to mental retardation and keratomalacia) are the main hazards of infants prematurely deprived of their mother’s milk and fed on inadequate substitutes in unhygienic conditions [16–18]. In Ethiopia, the proportion of mothers who still breastfeed their infants are considerably low [19]. A study conducted in Southwest Ethiopia showed that the proportion of exclusive breastfeeding was 37.9% at the end of the first month which dropped to 9.9% at the age of 6 months [20]. These mothers started using commercially processed, packed infant formula milk and natural cow’s milk as a substitution for breast milk. Another study conducted in similar parts of Ethiopia showed that mothers introduced supplementary foods (cow’s milk and formula milk) at an average of 2 months [21]. The tendency to use the formula-feeding increased in relation to child or infant increasing age. About 17% of the infants under the age of 3 months were offered formula and it increased to 69% in infants from 4 to 6 months [11].

According to the 2011 Ethiopian Demographic Health Survey (EDHS, 2011), 48% of mothers formula feed their children during the first 6 months after birth. Formula-feeding was 30% among the age of 0–1 month, it was 45% between 2 and 3 months and it increased to 68% in the infants from 4 to 5 months [19]. Even though early and timely breastfeeding is one of the key components of primary health care in Ethiopia, a wide range of traditional and cultural beliefs related to infant formula-feeding practices are documented even after implementations of the national infant and young child feeding recommendations [19]. However, there are no studies which documented formula-feeding practice and factors associated with formula-feeding practices in the study area particularly in a comparative way. Moreover, the magnitude of formula-feeding is not well studied in relation to the urban and rural basis. Thus, this study assessed the magnitude of formula-feeding practice and associated factors among urban and rural mothers in the Jimma Zone, specifically between the urban and rural part.

## Methods

### Study setting and sampling

A cross-sectional community-based comparative study was carried out in the Jimma zone Oromia region in South West Ethiopia, 327 km removed from Addis Ababa, based on the figure published by the Central Statistical Agency (CSA) in 2007. The Jimma zone has an estimated total population of 2,800,000 people [22]. A total of 714 mothers having children aged 0 to 6 months were interviewed from November 2015 to January 2016.

The sample size was obtained using a sample size calculation for a comparative cross-sectional study. Residence (being urban or rural) was considered as the main factor for practicing formula-feeding and was used for sample size determination. The sample size was calculated based on the following assumptions: A prevalence of mothers who practiced formula-feeding to their children in the rural area to be 50% (no previous study in the study setting);a prevalence of mothers who practiced formula-feeding to their children in the urban area to be 35% (from the previous similar study (21)); a power of 80% (0.841), a 5% type I error, a 5% non response rate and a design effect of 2 for the multistage nature of the sampling technique. Therefore, the final sample size was 714 with one to one ratio (357 mothers from each urban and rural area).

**Fig. 1:**
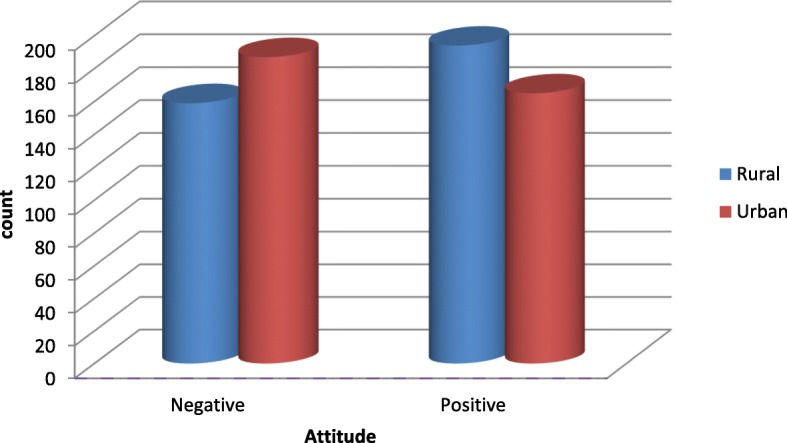
Sampling technique and procedure for formula-feeding practice in the Jimma zone, Oromia, January 2016

A multistage cluster sampling technique was used to select the respondents from 20 districts of the Jimma zone. Since the study units came from the Jimma zone to specific sub-districts, the first group was formed using district as a cluster (both from urban and rural), then based on the WHO recommendation, which is 30% of the districts (both from urban and rural) were selected through lottery method. In the second stage again 30% of sub-districts (both from urban and rural) was selected from each district. Sample size for each group was allocated according to the proportion to the number of mothers in the specific sub-districts. Finally, a simple random sampling technique was used in the respective sub-districts to select the study unit. After a list of mothers having children 0–6 months was identified, age identification numbers were taken from the registration book of health extension works in each sub-district. The data collection was continuous until the predetermined sample size obtained (Fig. 1).

This study included mothers who were a permanent resident of the selected sub-districts and had infants aged from 0 to 6 months; however, self-reporting mothers with any medical condition incompatible with breastfeeding including HIV positive were not included.

### Measurements

Data were collected using structured questionnaires which comprised of information on socio-demographic characteristics, knowledge, attitude, and practice regarding infant formula feeding (Additional file 1). The questionnaire was prepared in English after reviewing relevant literatures [20, 21]; translated to Afan Oromo and finally retranslated back to English by a person who can speak both languages. The questionnaire was pre-tested prior to the actual data collection. All necessary modifications were made to the questionnaire including terminologies and formatting based on the pretest findings. The supervisors checked each completed questionnaire and principal investigators monitored the overall quality of the data. In this study, formula-feeding practice was defined as the feeding of an infant less than 6 months old using a formula with a rubber nipple, cup and spoon and other materials on the end as a substitute for or supplement to breastfeeding and it was measured by a “Yes” (1) and “No” (2) question. The mothers who answered “Yes” to the question were considered a mother who feeds their child by formula-feeding.

Environmental or enabling factors are groups of a variable that affect the practice of formula-feeding by mothers. In this study, those variables were measured by availability and affordability of the formula milk. The variable was measured by “Yes” (1) and “No” (2).

Individual factors are those factors that include knowledge and perception towards formula feeding, maternity experience and attitude towards formula-feeding. In this study, knowledge and previous experience were measured using a “Yes” (1) and “No” (2), questioner and the attitude towards formula- feeding was measured by a Likert scale with three labels. The options were “agree” (3), “neutral” (2), and “disagree” (1). All attitude questions were summed up and treated as a continuous variable and those participants with the highest score were considered as having a favorable attitude towards formula-feeding questions.

### Data analysis

Data were entered to the Epi-info Version 3.5.3 and analyzed using SPSS (SPSS Inc. version 21.0, Chicago, Illinois). The socio-demographic and other information was stratified in to rural and urban categories and were descriptively presented by tables. Continuous variable such as age were expressed using mean ± SD and the categorical forms are presented in the tables. Bi-variate analysis was used to select the best predictor variables and those variables which showed a significant association at a *p*-value of < 0.25 were entered to the multiple logistic regression models and a *p*-value of < 0.05 was used to measure the significance in the final predictors of formula feeding. Strength and direction of the association were also presented using adjusted odds ratios (AOR) relative to the reference category and using 95% confidence levels.

## Results

### Socio-demographic characteristics of participants

From the overall sample size, 705 mothers (98.7%) responded to the questionnaire completely. Based on their living arrangements, 352 mothers (49.9%) were living in urban areas and 353 mothers (50.1%) were living in rural areas. The mean age of the respondents was 27.4 years (SD ± 4.7), with a minimum age of 16 years and a maximum age of 41 years. The mean age of the children was 3.5 months with a minimum of 1 month and a maximum of 6 months. Regarding the religion of the respondents, 597 mothers (84.7%) identified as Islam. Regarding educational status, 85 mothers (24.1%) living in the urban areas and 298 mothers (84.4%) living in rural areas were illiterate [Table 1].

**Table 1:** Frequency distribution of mothers by socio-demographic characteristics, Jimma zone, Oromia January 2016

Variables	Living arrangements		Total
	Rural (*N* = 353)	Urban (*N* = 352)	
	N[%]	N[%]	
Mother /Care taker age			
15–24	59 [16.7]	108 [30.7]	167
25–34	243 [68.8]	222 [63]	465
35–45	51 [14.5]	22 [6.25]	73
Mother educational status			
Illiterate	298 [84.4]	85 [24.1]	319
Read and write	55 [15.6]	59 [16.8]	80
		51 [14.5]	97
		56 [15.9]	96
		61 [17.3]	73
		40 [11.4]	40
Mother marital status			
Married	348 [98.6]	333 (94.6)	681
Widowed	0	13 [3.7]	13
Divorced	5 [1.4]	6 [1.7]	11
Religion			
Islam	320 [90.7]	277 [78.7]	597
Orthodox	16 [4.5]	59 [16.8]	75
Protestant	17 [4.8]	16 [4.5]	33
Ethnicity			
Oromo	321 [90.9]	304 [86.4]	625
Amhara	8 [2.3]	24 [6.8]	52
Other^a^	24 [6.8]	24 [6.8]	48
Mother/care taker occupation			
Farmer	164 [6.4]	30 [8.5]	194
Governmental employee	2 [0.6]	40 [11.4	42
House wife	178 [50.4]	227 [64.5]	405
Merchant	7 [2]	48 [11.6]	55
Other^b^	2 [0.6]	7 [2]	9

^a^kaffa, Gurage, Yem, Tigirian, Wolayita and dawuro; ^b^Daily laborers and housekeeper

### Maternity experience

On average, each mother gave birth to 3 children. A total of 309 mothers (87.5%) in the rural and 296 mothers (84.1%) in the urban area attended ANC. About 138 mothers (46.6%) in the urban area had four times ANC visit during their last pregnancy (Table 2).

**Table 2:** Maternity experiences of mothers in Jimma zone, Oromia January 2016

Variables	Living arrangements		Total
	Rural (*N* = 353)	Urban (*N* = 352)	
	N [%]	N [%]	
ANC Visit			
Yes	309 [87.5]	296 [84.1]	605
No	44 [12.5]	56 [15.9]	100
Number of ANC visit			
1	4 [1.3]	5 [1.7]	9
2	50 [16.2]	53 [18]	103
3	136 [44]	97 [32.7]	233
4	113 [36.6]	138 [46.6]	251
I do not know	6 [1.9]	3 [1]	9
PNC follow up			
Yes	248 [70.3]	270 (76.7)	518
No	105 [29.7]	82 [23.3]	187
Place of delivery			
Home	166 [47]	53 [15]	219
Governmental health facility	177 [50.1]	286 [81.2]	463
NGOs health facility	9 [2.6]	11 [3.2]	20
On the way to health	1 [0.3]	2 [0.6]	3
Facility			
Birth attendants			
Health professionals	183 [51.8]	300 [85.2]	483
Relatives/friends/neighbors	138 [39.1]	39 [11.1]	177
TBA/TTBA	32 [9.1]	13 [3.7]	45

*ANC* Antenatal care, *NGOs* None Governmental Organizations, *PNC* Postnatal care, *TBA* Traditional Birth Attendants, *TTBA* Trained Traditional Birth Attendants

### Awareness of mother/care taker on formula-feeding

The majority of the mothers both in the rural and urban areas heard about the effect of formula-feeding on the health of their child. Diarrhea was mentioned by the majority (231, 53.6%) of the mothers as a health problem caused by formula feeding, followed by an intestinal parasite (107, 24.8%) and tonsil (93, 21.6%). About 85 mothers (24.1%) in the urban area and 91 mothers (25.8%) in the rural area have received health education about formula-feeding during their last pregnancy.

### Attitude towards formula feeding

**Fig. 2:**
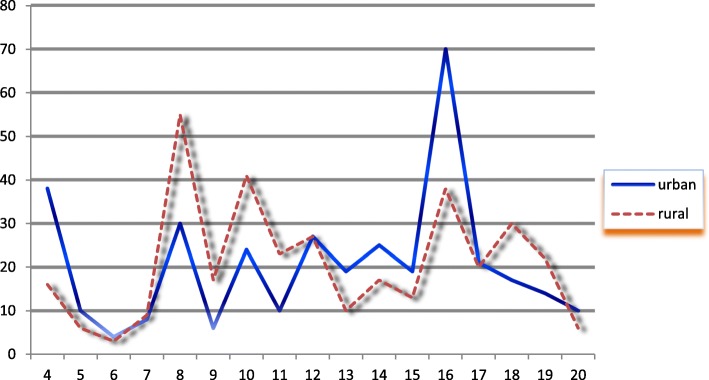
Mothers’ attitude towards formula-feeding in the Jimma zone, Oromia, January 2016

About 178 mothers (50.4%) in the rural areas and 176 mothers (50%) in the urban areas disagreed with the idea that formula-feeding ensures optimal health for the baby. In general, 159 mothers (45%) in the rural area and 187 mothers (53.2%) in the urban areas had a negative attitude towards formula-feeding (Table 3), see also Fig. 2.

**Table 3:** Attitude towards formula-feeding among mothers in Jimma Zone, Oromia January, 2016

Item	Responses						Total
	Rural (*N* = 353)			Urban (*N* = 352)			
	Disagree	Neutral	Agree	Disagree	Neutral	Agree	
	N [%]	N [%]	N [%]	N [%]	N [%]	N [%]	
Formula-feeding ensures optimal health for the baby.	178 [50.4]	58 [16.4]	117 [33.2]	176 [50]	57 [16.2]	119 [33.8]	705
Formula-feeding can causes excessive weight gain in baby.	176 [49.9]	76 [21.5]	101 [28.6]	194 [55.1]	55 [15.6]	103 [29.3]	705
Formula-feeding is more convenient than breastfeeding.	191 [54.1]	76 [21.5]	86 [24.4]	245 [69.6]	44 [12.5]	63 [17.9]	705
Formula-feeding ensures optimal health for the mother.	168 [47.6]	40 [11.3]	145 [41.1]	150 [42.6]	57 [16.2]	145 [41.2]	705
Formula-feeding babies tend to be fed less frequently.	195 [55.2]	40 [11.3]	118 [33.5]	173 [49.2]	79 [22.4]	100 [28.4]	705
The nutritional benefit of breast milk lasts only until the baby is weaned from breast milk.	222 [62.9]	38 [10.8]	93 [26.3]	180 [51.1]	47 [13.4]	125 [35.5]	705

### Social pressure

The pressure of religious leaders, mothers-in-law, grandmothers/elderly and husbands’ relatives was significantly associated with the decision of the mother to formula-feed her children. Social pressure scores were analyzed as a continuous variable with possible values ranging from 3 to 20 for both living arrangements. The mean score of social pressure for mothers living in the urban area was 12.4 (±4.6) and the mean score for mothers living in the rural areas was 12.3 (±4.3).

### Practice on formula-feeding

Majority, 218 mothers (61.9%) in the urban area were practicing formula-feeding for their child. On the other hand, only 115 mothers (32.6%) in the rural areas practiced formula-feeding for their child. Among the rural dwellers who practiced formula-feeding (115), the frequency of formula-feeding was one to four times a day in 85 mothers (73.9%) (Table 4).

**Table 4:** Formula-feeding practice of mothers in the Jimma zone, Oromia, January 2016

Variables	Living arrangements		Total
	Rural *N* = 353	Urban *N* = 352	
	N [%]	N [%]	
Formula feed			
Yes	115 [32.6]	218 [61.9]	333
No	238 [67.4]	134 [38.1]	372
Frequency of formula feeding			
Unknown	7 [6.1]	44 [20.2]	51
1 to 4 times	85 [73.9]	109 [50]	194
5 and above	23 [20]	65 [29.8]	88
Food introduced first			
Cow milk	83 [72.2]	64 [29.4]	147
Infant formula	8 [6.9]	98 [45]	106
Fruits juice	8 [6.9]	45 [20.6]	53
Tea	16 [14]	2 [0.9]	18
Other^a^	0	9 [4.1]	9
Age at first baby start formula feeding			
1–6 months	33 [28.7]	113 [51.8]	146
7 and above months	82 [71.3]	105 [48.2]	187

^a^Flax seed, fenugreek, and oats juice

### Bivariate logistic regression analysis of formula-feeding practice among mothers living in the urban and rural areas

The bivariate analysis showed that women in the rural areas were 3.36 times more likely to formula-feed their child than women living in the urban areas. The observed difference was statistically significant [COR = 3.36, (95%CI2.41–4.58], *P* = 0.00] (Table 5).

**Table 5:** Comparison of formula-feeding practice among mothers living in the rural and urban areas in the Jimma zone, January, 2016

Variable	Formula-feeding practice		Crude OR[95% CI]
	Yes	No	
	N [%]	N [%]	
Living arrangements			
Rural	115 [34.5]	238 [64]	3.36[2.41–4.58]*
Urban	218 [65.5]	134 [36]	1.00
Total	333 [100]	372 [100]	

*CI* Confidence interval, *COR* Crude odds ratio; 1: referent; *statisticaly significant at *P*<0.05

All variables with statistical significance have been made in bold face

### Predictors of formula-feeding practice among mothers living in the rural areas

In order to determine the influence of each predictor on the formula-feeding practice, all variables that become significant at the bivariate analysis were included in multivariate logistic regression for further analysis. Mothers who heard about the health effect of formula-feeding were 41% less likely to formula-feed their child. Being attended by traditional birth attendants and relative or friends increases the likelihood of formula-feeding by 48 and 97% respectively [AOR = 6.892, 95%CI = 1.103–43.061, AOR = 8.702, 95% CI = 1.437–52.709].

For social pressure among mothers living in the rural areas, per a unit increase in a total score of social pressure, the odds of formula-feeding reduces by 0**.**83 and the observed difference was statistically significant [AOR, 95%CI .83(.78–.89)] (Table 6).

**Table 6:** Bivariate and multivariate analysis of formula-feeding practice among mothers living in the rural areas, Jimma zone, January, 2016

Variables	Formula-feeding practice		COR(95% CI)	AOR(95% CI)
	No (*N* = 238)	Yes (*N* = 115)		
	N [%]	N [%]		
Mother /Care taker age				
15–24	45 [76.3]	14 [23.7]	1.216[.515–2.875]	
25–34	156 [64.2]	87 [3.8]	.678[.348–1.32]	
35–45	37 [72.5]	14 [27.5]	1.00	
Sex of the infant				
Male	121 [68]	57 [32]	1.05[.67–1.64]	
Female	117 [66.9]	58 [33.1]	1.00	
Educational status of Mother				
Illiterate	109 [36.6]	189 [63.4]	.212 [.088–.512]	.186[.072–.481]
Read and write	6 [10.9]	49 [89.1]	1	
Ethnicity				
Oromo	216 [67.3]	105 [32.7]	1.00	
Amhara	4 [50]	4 [50]	.486 [.119–1.82]	
Others	18 [75]	6 [25]	1.45 [.562–3.78]	
Mother occupation				
Farmer	124 [75.6]	40 [24.4]	1.00	1.00
House wife	105 [59]	73 [41]	.464 [.29–.739]*	0.405[.243-.675]
Governmental	9 [81.8]	2 [18.2]	1.452 [.30–6.99]	0.583[.108–3.159]
Employee				
ANC visit				
Yes	210 [68]	99 [86.1]	1.00	
No	28 [11.8]	16 [13.9]	.825 [0.427–1.595]	
PNC visit				
Yes	171 [71.8]	73 [63.5]	1.00	
No	67 [28.2]	42 [36.5]	.681 [.424–1.093]	
Place of delivery				
Home	131 [55.3]	35 [30.4]	2.825 [1.761–4.531]*	399[.069–2.326]
Health facility	106 [44.7]	80 [69.6]	1.00	
Birth attendants				
Health worker	131 [55.3]	80 [69.5]	1.00	1.00
TTBA/TBA	21 [8.8]	8 [7]	2.039 [.858–4.893]	**6.892[1.103–43.061]** *
Relative/Friends.	114 [39.9]	27 [23.5]	3.279 [1.967–5.468]*	**8.702[1.437–52.709]** *
Awareness about formula feeding				
Yes	172 [72.3]	50 [43.5]	1.00	
No	66 [27.7]	65 [56.5]	3.38 [2.127–5.395]*	**.401[.237–.677]** *
Attitude towards formula feeding				
Positive	125 [53.4]	67 [58.3]	1.00	
Negative	111 [46.6]	48 [41.7]	1.220 [.778–1.913]	
Social pressure			0.831 [.784–.881]*	.837[.780–.898]*

*ANC* Antenatal Care, *AOR* Adjusted odds ratio, *CI* Confidence interval, *COR* Crude odds ratio, *PNC* Postnatal care, *TBA* Traditional birth attendant, *TTBA* Trained traditional birth attendant; 1: referent; *significant at *p* value< 0.05

All variables with statistical significance have been made in bold face

### Predictors of formula-feeding practice among mothers living in the urban areas

In the bivariate analysis, educational status of the mother, place of delivery, awareness about the health effect of formula feeding, attitude towards formula-feeding and social pressure were significantly associated with formula-feeding practice at *P* value < 0.05. By the final model, the educational status of the mother was significantly associated with formula-feeding practice. The likelihood of formula-feeding increases by 39% among illiterate mothers [AOR = 3.39, 95%CI 1.41–8.17]. Awareness about the health effect of formula-feeding reduces the likelihood of formula-feeding by 0.29 among mothers living in the urban areas [AOR = .294, 95%CI .167–.517].

Regarding the attitude towards formula-feeding, having negative attitude increases the probability of not formula-feeding by 74% [AOR = 2.749, 95%CI 1.626–4.648]. A unit decline in social pressure reduces the risk of formula-feeding by 0.925[AOR = .925, 95%CI, .875–.977] (Table 7).

**Table 7:** Bi and multivariate analysis of formula-feeding practice among mothers living in the urban areas, Jimma zone, January, 2016

Variables	Formula-feeding practice		COR (95% CI)	AOR (95% CI)
	No (*N* = 134)	Yes (*N* = 218)		
	N [%]	N [%]		
Mother /Care taker age				
15–24	42 [38.9]	66 [61.1]	2.164 [.742–6.305]	
25–34	87 [39.2]	135 [60.8]	2.191 [.78–6.155]	
35–45	5 [22.7]	17 [77.3]	1.00	
Sex of child				
Male	64 [37.2]	108 [62.8]	.931 [.602–1.432]	
Female	70 [33.9]	110 [61.1]	1.00	
Educational status of the Mother				
Illiterate	44 [51.8]	44 [48.2]	2.50 [1.126–5.567]*	3.39 [1.410–8.179]*
Read and write	18 [30.5]	41 [69.5]	1.024 [.427–2.456]	1.029 [.38–2.77]
Grade [1–4]	21 [41.2]	30 [58.8]	1.633 [.680–3.924]	1.705 [.662–4.388]
Grade [5–8]	16 [28.6]	40 [71.4]	.933 [.383–2.275]	.872 [.336–2.261]
Grade [9–12]	23 [37.7]	38 [62.3]	1.412 [.603–3.310]	1.397 [.563–3.466]
Above 12	12 [30]	28 [70]	1.00	1.00
Religion				
Islam	106 [38.3]	171 [61.7]	1.00	
Orthodox	19 [32.2]	40 [67.8]	.766 [.422–1.393]	
Protestant	9 [56.2]	7 [43.8]	2.074 [.750–5.735]	
Ethnicity				
Oromo	118 [38.8]	186 [61.2]	1.00	
Amhara	6 [25]	18 [75]	.525 [.203–1.362]	
Other	18 [75]	6 [25]	1.126 [.482–2.618]	
Mother occupation				
Farmer	14 [46.7]	16 [53.3]	1.00	
House wife	80 [35.5]	147 [64.8]	.622 [.289–1.340]	
Other	40 [42.1]	55 [57.9]	.831 [.346–1.896]	
ANC visit				
Yes	111 [37.5]	185 [62.5]	1.00	
No	23 [41.1]	33 [58.9]	1.162 [.649–2.08]	
PNC visit				
Yes	105 [38.9]	165 [61.1]	1.00	
No	29 [35.4]	53 [64.6]	.860 [.514–138]	
Place of delivery				
Home	27 [50.9]	26 [49.1]	1.844 [1.02–3.3]*	1.857 [.954–3.61]
Health facility	107 [36]	190 [64]	1.00	
Birth attendants				
Health worker	110 [36.7]	190 [63.3]	1.00	
TTBA/TBA	7 [53.8]	6 [46.2]	2.015 [.661–6.148]	
Relative/Friends.	17 [43.6]	22 [56.4]	1.335 [.680–2.622]	
Awareness about formula feeding				
Yes	95 [45.5]	114 [54.5]	1.00	1.00
No	639 [27.3]	104 [72.7]	.450 [.285–.711]*	.294 [.167–.517]*
Attitude towards formula feeding				
Positive	44 [2.7]	121 [73.3]	1.00	1.00
Negative	90 [48.1]	97 [51.9]	2.55 [1.629–3.9]*	2.75 [1.626–4.6]*
Social pressure			0.948 [.90–.993]*	.925 [.875–.977]*

*ANC* Antenatal Care, *OR* Odds ratio, *AOR* Adjusted odds ratio, *CI* Confidence interval, *COR* Crude odds ratio, *PNC* Postnatal care, *TBA* Traditional birth attendant, 1: referent; *significant at *p* value< 0.05

All variables with statistical significance have been made in bold face

## Discussion

Formula-feeding is becoming a common practice in various parts of Ethiopia both in urban and rural areas due to various socio-cultural reasons. In this study almost half of the mothers (47.2%) practiced formula-feeding for their babies, of whom 65.5 and 34.5% of them were mothers living in the rural and urban areas respectively.

Even though formula-feeding practice was not specifically assessed and it is difficult to make comparisons, non-human milk feeding was 35% (lower than the current finding) in the general population as to the study conducted in Uganda [23]; non-exclusive breastfeeding was also found to be 57% in Malawi [24], 49.4% in Sorro district, southern Ethiopia [25], 28% in Offa district, southern Ethiopia [26] and 30.1% in Hawassa town, Southern Ethiopia [27].

Being aware of the health effect of formula-feeding and social pressures was significantly associated with the formula-feeding practice among mothers in both rural and urban settings. This indicates that anyone respected by mothers such as religious leaders, mothers-in-law or grandmothers, husbands and other close relatives have a significant impact on the decision of mothers to formula-feed their child or not.

In addition, mothers who have less awareness about the health effect of formula-feeding on their children turned out to have increased formula-feeding practices. Supporting this finding, a study conducted in Eastern Ethiopia found that mothers whose knowledge is low on infant and child feeding practices and those with no access to healthcare facility were more likely to practice non-exclusive breastfeeding, implicating the low awareness on the health effect of formula-feeding [28]. On the other hand, a study conducted in the Offa district, southern Ethiopia supported this finding; those mothers who were able to read and write and those who were aware of exclusive breastfeeding were 1.1 and 6 times more likely to exclusively breastfeed their baby [26]. Therefore, the knowledge of the mothers could be a prominent reason of formula-feeding practice during the early infancy; that is why illiterate mothers are 3.39 times more likely to formula-feed their infants. Moreover, their level of knowledge might have affected their attitude towards formula-feeding (AOR: 2.749; 95% CI: 1.626–4.648).

Regarding the delivery attendant, delivery attended by TBA/relatives/friends/neighbor was an increasing factor for formula-feeding among mothers in the rural areas. This may be due to information given to mothers following delivery about exclusive breastfeeding and the health impact of formula-feeding. In line with this finding, no antenatal care (AOR: 2.6; 95% CI: 1.64–4.10) during the last pregnancy and no postnatal care (AOR: 1.9; 95% CI: 1.19–3.04) were significant factors for non-exclusive breastfeeding [25]. According to the study conducted in Hawassa South Ethiopia, mothers who delivered at a health care facility [AOR: 8.8; CI: 5.04–15.4) were more likely to practice exclusive breastfeeding [27]. This means that home delivery could be a major contributor for non-exclusive breastfeeding large fraction of the infants (35.1%) took liquid forms of food that may define formula-feeding [27].

## Conclusion

Nearly half of the mothers in the study area, majorly mothers living in urban areas, practice formula-feeding for their infant. Being aware about the health effect of breastfeeding and having minimal social pressure decreases the likelihood of formula-feeding practice among mothers in both settings. Illiterate mothers and mothers who had a positive attitude towards formula-feeding were more likely to formula feed their child in the urban areas. A birth attended by relatives/friends/traditional birth attendants’ increases the likelihood of formula-feeding among mothers living in the rural area. Therefore, Zonal and district health offices and organizations working on maternal and child health should work to create awareness on the health effect of formula-feeding on infants; design tailored communication to change the attitude of the mothers on formula-feeding and they should work to reduce the influence of social pressure on mothers, by creating awareness among the social cycle could reduce formula-feeding practices in the study area.

## Supplementary information

**Additional file 1.** Questionnaire: English version

## Data Availability

The datasets used and/or analysed during the current study available from the corresponding author on reasonable request.

## Abbreviations

ANC: Antenatal Care
DHS: Demographic and Health Survey
FMOH: Federal Ministry of Health
HEW: Health Extension Worker
MCH: Maternal Child Health
NGO: Non-Governmental Organization PNC Post-Natal Care
TBA: Traditional Birth Attendants
TTBA: Trained Traditional Birth Attendants
USA: United State of America
WHO: World Health Organization
