# Supplementary material for: Formula-feeding practice and associated factors among urban and rural mothers with infants 0–6 months of age: a comparative study in Jimma zone Western Ethiopia
Source: BMC Pediatr. 2019 Nov 4;19:408. doi: 10.1186/s12887-019-1789-8 (PMC6827217; doi:10.1186/s12887-019-1789-8)
Supplement: Supplementary file 1 — Additional file 1. Questionnaire: English version [file 12887_2019_1789_MOESM1_ESM.docx]

**Annex III. English version Questionnaire**

Formula-Feeding Practice and Associated Factors among Urban and Rural Mothers with infants 0-6 months of age: A comparative study in Jimma Zone Western Ethiopia.

**Instructions:** Follow the instructions and guides under the questions to fill the questionnaire.

**Part 1: Socio – demographic characteristics**

| S.No | | | Question | Response |
| --- | --- | --- | --- | --- |
| 001 | | | Living arrangement of the mother | 1. Urban 2. Rural |
| 002 | | | How old are you? Age(in completed year) | _______________ |
| 003 | | | What is your current marital status? | 1. Single 2. Married 3. Divorced 4. Widowed |
| 004 | | | What is your educational level? (Based on last completed grade level) | 1. Illiterate 2. read and write only 3. Elementary (1-8 grades) 4. Secondary (9-10 grades) 5. Preparatory (11-12 grades) 6. College and above |
| 005 | | | What is your husband’s educational level?(Based on last completed grade level) | 1. unable to write and read 2. read and write only 3. Elementary (1-8 grades) 4. Secondary (9-10 grades) 5. Preparatory (11-12 grades) 6. College and above |
| 006 | | | What religion do you follow? | 1. Orthodox 2. Muslim 3. Protestant 4. Catholic   5. Other Specify__________________ |
| 007 | | | Your family size (in number ) | ___________ |
| 008 | | | What is your current occupation? | 1. House wife 2. Private business 3. Employee 4. Student 5. Other, Specify __________________ |
| 009 | | | What is your husband’s current occupation? | 1. Private business 2. Employee 3. Farmer 4. Student 5. Other, Specify____________ |
| 010 | | | What is your Ethnicity? | 1. Oromo 2. Amhara 3. Others, Specify______________ |
| 011 | | | What is the average monthly income of the household? | _________________in birr |
| **Part II Maternity Experiences** | | | | |
| 012 | | | Did you have ANC care follow-up visits in any health facility while you were pregnant for the last child? | 1. Yes 2. No If No, go to Q15 |
| 013 | | | How many ANC follow-up visits did you attend in your last child?(in number) | _________________ |
| 014 | | | Were you counseled /advised about breastfeeding in your ANC follow-up visits? | 1. Yes 2. No |
| 015 | | | Where did you give birth to your last child? | 1. Home 2. Government health center 3. Government hospital 4. Private health facility   5.Other, specify__________ |
| 016 | | | Who were your assistants or Birth attendants | 1. Health professionals 2. Relatives/friends/neighbors 3. Traditional birth attendants 4. Others, specify__________ |
| 017 | | | Were you counseled/advised about nutrition including breast feeding during delivery? | 1. Yes 2. No |
| 018 | | | Following your last delivery did you have PNC follow-up visit? | 1. Yes 2. No If No, go to Q21 |
| 019 | | | How many PNC follow-up visits did you have? | (__________) |
| 020 | | | Were you counseled/advised about the importance of infant nutrition including breast feeding in your PNC follow-up visits? | 1. Yes 2. No |
| **III: Knowledge about formula-feeding practice** | | | | |
| 021 | | Have you heard the term formula-feeding**?** | | 1. Yes 2. No If No, go to Q30? |
| 022 | | From where did you get this information? | | 1. Radio 2. TV 3. Health professionals 4. Friends 5. Other specify____________ |
| 023 | | What does it mean the term formula-feeding? | | 1. Good nutrition is critical for physical and brain development of the baby starting from the first hours to 6 months of the child  2. doesn’t know |
| 024 | | Is good nutrition mandatory for brain and physical development of your child? | | 1. Yes 2. No if no go to Q32 |
| 025 | | When do you think good nutrition is critical for brain development of your child? | | 1. From child birth up to 6 months 2. From child birth up to two years 3. At any age there is brain development 4. I do not know |
| 026 | | Is brain damage due to malnutrition in the first 6 months of life is reversible? | | 1. Yes 2. No 3. I do not know |
| 027 | | What is the advantage/use of breastfeeding to your child? | | 1. Best diet for newborn 2. Prevents disease 3. Bonds mother and child 4. Grows best   5. Other, specify _________ |
| 028 | | How soon after child birth should  Breast feeding was started? | | 1. Within one hour of delivery 2. Within 6 hours of delivery 3. After 6 hours but within 24 hours of delivery 4. After 24 hours but within 48 hours of delivery   5.Other, specify |
| 029 | | How frequent should a child be breastfeed? | | 1. Whenever the child wants 2. Whenever the mother wants 3. On schedule   4. Other, specify _________ |
| 030 | | How long should child be only breastfeed? (in months) | | (_______) month |
| 031 | | What other additional foods or drinks  Should a child be fed from birth up to six months in addition to breast milk? | | 1. Nothing 2. Plain water 3. Water-sugar/salt solutions 4. Cow’s milk 5. Formula milk 6. Butter   5.Other, specify _________ |
| 032 | | Does lack of hygiene can affect nutritional status of your child? | | 1.Yes  2. No  3. I do not know |
| 033 | | What should people in the community do to maintain proper hygiene? | | 1. Washing hands with soap before feeding a child or preparing food 2. Washing hands with soap after using a latrine 3. Each household must have a latrine 4. Keeping water and soap for washing hands at the latrine 5. Avoiding stagnant water around the home 6. Other specify |
| **Attitude about formula-feeding practice** | | | | |
| 034 | | Formula-feeding ensures optimal health for Baby. | | 1. Strongly agree  2. Agree  3. Neutral  4. Disagree  5. Strongly disagree |
| 035 | | Formula-feeding can causes excessive weight gain in baby. | | 1. Strongly agree  2. Agree  3. Neutral  4. Disagree  5. Strongly disagree |
| 036 | | Formula-feeding is more convenient than breastfeeding. | | 1. Strongly agree  2. Agree  3. Neutral  4. Disagree  5. Strongly disagree |
| 037 | | Formula-feeding ensures optimal health for mother | | 1. Strongly agree  2. Agree  3. Neutral  4. Disagree  5. Strongly disagree |
| 038 | | Formula-feeding babies tend to be fed less frequently. | | 1. Strongly agree  2. Agree  3. Neutral  4. Disagree  5. Strongly disagree |
| 039 | | The nutritional benefit of breast milk last only until the baby is weaned from breast milk. | | 1. Strongly agree  2. Agree  3. Neutral  4. Disagree  5. Strongly disagree |
| 040 | | Dose the father support/encourage you to Formula-feed your child? | | 1. Yes 2. No |
| 041 | | Do other family members support/ encourage you to Formula-feed your child? | | 1. Yes 2. No |
| **Practice questions about formula feeding** | | | | |
|  | Did you fed your child any formula feeding | | | 1. Yes  2.No |
| 042 | How many months did you only breastfeed your child without adding any other foods or drinks starting from birth? | | | (__________) months |
| 043 | What did you fed your child the day he/she has born | | | 1. Fresh butter 2. Breast milk 3. Formula milk 4. Water 5. Caw milk 6. Fruits juice 7. Tea 8. Other specify__________________ |
| 044 | Age at first baby start formula-feeding | | | - 1. 1-24hours   2. 25-72hours   3. 1-6 Months   4. 6 and above months   5. Don’t know |

**Thank you for your responses and the time you spent!!**

| Variables | | | Living arrangements | | Total |
| --- | --- | --- | --- | --- | --- |
|  |  |  | Rural (N=353) | Urban (N=352) |  |
|  |  |  | N[%] | N[%] |  |
| Mother /Care taker age | | | | | |
|  | 15-24  25-34  35-45 | | 59[16.7]  243[68.8]  51[14.5] | 108[30.7]  222[63]  22[6.25] | 167  465  73 |
| Mother educational status | | |  |  |  |
|  | | Illiterate  Read and write | 298 [84.4]  55 [15.6] | 85[24.1]  59[16.8]  51[14.5]  56[15.9]  61[17.3]  40[11.4] | 319  80  97  96  73  40 |
| Mother marital status | | |  |  |  |
|  | Married  Widowed  Divorced | | 348 [98.6]  0  5[1.4] | 333 (94.6)  13[3.7]  6[1.7] | 681  13  11 |
| Religion | | |  |  |  |
|  | Islam  Orthodox  Protestant | | 320[90.7]  16[4.5]  17[4.8] | 277[78.7]  59[16.8]  16[4.5] | 597  75  33 |
| Ethnicity | | |  |  |  |
|  | Oromo  Amhara  Other* | | 321[90.9]  8[2.3]  24[6.8] | 304[86.4]  24[6.8]  24[6.8] | 625  52  48 |
| Mother/care taker occupation | | |  |  |  |
|  | Farmer  Governmental employee  House wife  Merchant  Other** | | 164[6.4]  2[0.6]  178 [50.4]  7[2]  2[0.6] | 30[8.5]  40[11.4  227[64.5]  48[11.6]  7[2] | 194  42  405  55  9 |
